# The peptide semax affects the expression of genes related to the immune and vascular systems in rat brain focal ischemia: genome-wide transcriptional analysis

**DOI:** 10.1186/1471-2164-15-228

**Published:** 2014-03-24

**Authors:** Ekaterina V Medvedeva, Veronika G Dmitrieva, Oksana V Povarova, Svetlana A Limborska, Veronika I Skvortsova, Nikolay F Myasoedov, Lyudmila V Dergunova

**Affiliations:** 1 Human Molecular Genetics Department, Institute of Molecular Genetics, Russian Academy of Sciences, Moscow, Russian Federation; 2 Institute of Cerebrovascular Pathology and Stroke, Pirogov Russian National Research Medical University, Moscow, Russian Federation; 3 Faculty of Medicine, Moscow State University n/a M.V. Lomonosov, Moscow, Russian Federation

**Keywords:** Semax, Pro-Gly-Pro, Focal cerebral ischemia, Expression Beadchip gene array, Gene expression, Immune cells, Immunoglobulins

## Abstract

**Background:**

The nootropic neuroprotective peptide Semax (Met-Glu-His-Phe-Pro-Gly-Pro) has proved efficient in the therapy of brain stroke; however, the molecular mechanisms underlying its action remain obscure. Our genome-wide study was designed to investigate the response of the transcriptome of ischemized rat brain cortex tissues to the action of Semax *in vivo*.

**Results:**

The gene-expression alteration caused by the action of the peptide Semax was compared with the gene expression of the “ischemia” group animals at 3 and 24 h after permanent middle cerebral artery occlusion (pMCAO). The peptide predominantly enhanced the expression of genes related to the immune system. Three hours after pMCAO, Semax influenced the expression of some genes that affect the activity of immune cells, and, 24 h after pMCAO, the action of Semax on the immune response increased considerably. The genes implicated in this response represented over 50% of the total number of genes that exhibited Semax-induced altered expression. Among the immune-response genes, the expression of which was modulated by Semax, genes that encode immunoglobulins and chemokines formed the most notable groups.

In response to Semax administration, 24 genes related to the vascular system exhibited altered expression 3 h after pMCAO, whereas 12 genes were changed 24 h after pMCAO. These genes are associated with such processes as the development and migration of endothelial tissue, the migration of smooth muscle cells, hematopoiesis, and vasculogenesis.

**Conclusions:**

Semax affects several biological processes involved in the function of various systems. The immune response is the process most markedly affected by the drug. Semax altered the expression of genes that modulate the amount and mobility of immune cells and enhanced the expression of genes that encode chemokines and immunoglobulins. In conditions of rat brain focal ischemia, Semax influenced the expression of genes that promote the formation and functioning of the vascular system.

The immunomodulating effect of the peptide discovered in our research and its impact on the vascular system during ischemia are likely to be the key mechanisms underlying the neuroprotective effects of the peptide.

## Background

Ischemic brain stroke is one of the major contributors to mortality and disability worldwide. As the result of a critical reduction of blood flow in the brain, it causes massive loss of neurons and leads to the formation of the necrotic core and the penumbra zone [1].

One of the drugs that is effectively employed currently in cerebral stroke therapy is the Semax (Met-Glu-His-Phe-Pro-Gly-Pro), which is a synthetic peptide consisting of a fragment of ACTH(4–7) and the C-terminal tripeptide Pro-Gly-Pro (PGP). Studies have shown that Semax promotes the survival of neurons during hypoxia [2] and glutamate neurotoxicity [3]. It also shows neuroprotective properties and contributes to mitochondrial stability under stress induced by the deregulation of calcium ion flow [3]. The action of Semax causes the inhibition of nitric oxide synthesis [4], improves the trophic supply of the brain [5], and protects the nervous system effectively against diseases of the optic nerve [6]. This peptide also possesses nootropic activity [7].

However, the molecular mechanisms underlying the action of Semax remain unclear. We have previously shown the effect of Semax on the expression of genes that encode neurotrophic factors and their receptors in an experimental model ischemia in the rat brain [8,9].

This genome-wide study was performed to elucidate the transcriptome response of the ischemized focal tissues of the rat brain to the action of Semax in vivo. The main task of our study was to identify genes with an altered expression that accounts for the positive effect exerted by Semax in the treatment of patients with ischemic stroke [10,11].

## Results

### Semax-induced increase and decrease in gene expression

The genome-wide expression changes induced by Semax in rat brain cortex tissues damaged by focal ischemia were studied using the genome-wide RatRef-12 Expression BeadChip (Illumina, USA), which contains 22,226 genes, according to NCBI. Data on the gene expression changes induced by the peptide were compared with the gene expression levels in the “ischemia” group at 3 and 24 h after pMCAO.

**Figure 1:**
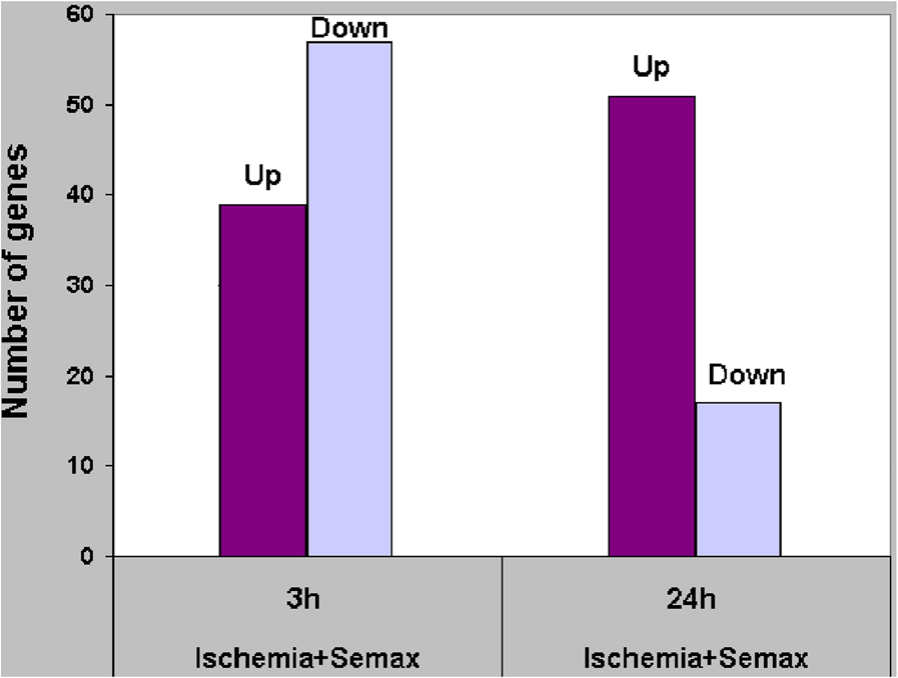
**Genes that were up- and downregulated.** The x-axis shows the condition of the experiment and time after pMCAO. The y-axis represents the number of genes that exhibited changed expression in these conditions. The cut-off of gene-expression changes was 1.50.

The largest number of genes (96) that exhibited altered expression (cut-off 1.50) in response to Semax administration was detected 3 h after the onset of ischemia (Additional file 1); moreover, the amount of the genes with decreased expression was insignificantly larger than that of those with increased expression (Figure 1). Semax altered the expression of 68 genes 24 h after occlusion (Additional file 2): the expression of 51 genes was increased and the amount of genes with decreased expression was considerably lower than that observed at 3 h after the onset of ischemia.

Note that different gene groups exhibited Semax-induced alteration of expression at 3 h and 24 h. The overlapping group comprised only 10 genes with responses to the peptide that were contradictory (Table 1).

**Table 1:** Comparison of genes that exhibited Semax-induced alteration of expression levels after pMCAO

**Gene symbol**	**Ischemia + semax 3 h**		**Ischemia + semax 24 h**		**ENTREZ GENE_ID**
	**lg (Ratio)**	***P*-value**	**lg (Ratio)**	***P*-value**	
*RGD1562905*	0.58	1.1E-04	−0.53	2.1E-34	292539
*Adamts1*	0.34	5.2E-11	−0.25	7.5E-09	79252
*Zfp36*	0.24	5.5E-03	−0.16	2.0E-04	79426
*Ptprcap*	−0.28	2.3E-03	0.29	9.8E-08	499300
*Ccdc53*	−0.29	1.3E-06	0.18	4.6E-03	299707
*RT1-Ba*	−0.29	3.1E-07	0.36	1.5E-15	309621
*Cd74*	−0.30	1.6E-09	0.21	5.3E-06	25599
*RT1-A1*	−0.30	7.6E-10	0.29	2.8E-11	24973
*H2-Ea*	−0.31	4.1E-10	0.15	7.3E-04	294269
*Igh-1a*	−0.79	2.6E-30	0.47	3.5E-26	299352

The table shows the genes that exhibited significantly changed expression under Semax treatment at both time points (3 and 24 h). The logarithmic transformed value visually represents the symmetric changes in expression levels: negative value indicates decrease and positive value – increase of the gene expression level. The cut-off of gene-expression changes was 1.40. *Ratio* – relative expression level.

### Molecular functions of the protein products of genes with altered expression under Semax treatment

**Figure 2:**
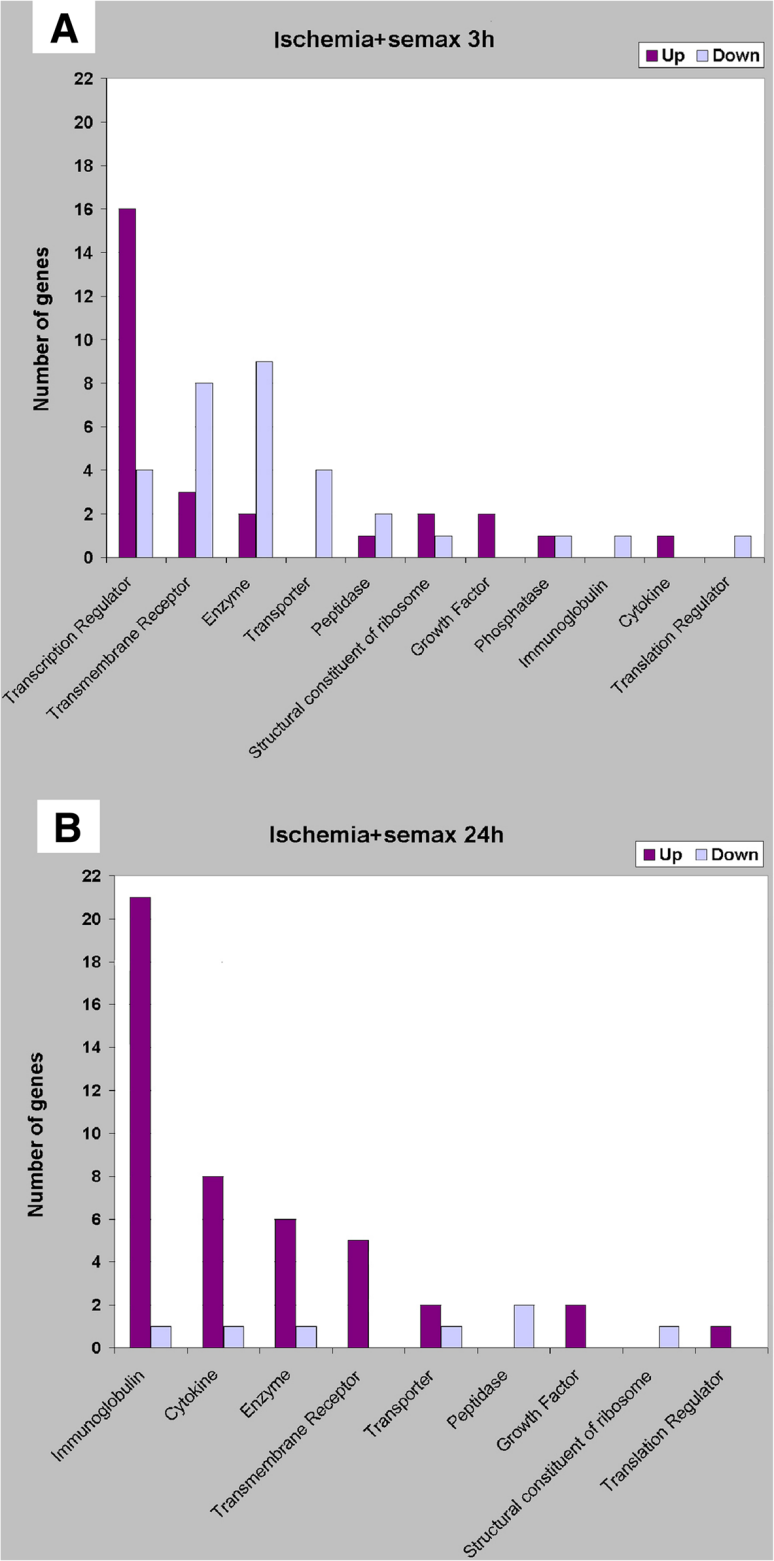
**Molecular functions associated with the up- and downregulated genes.** The x-axis shows the categories of molecular functions. The y-axis represents the number of genes associated with selected cellular functions. The genes that were upregulated are indicated by dark columns, whereas the genes that were downregulated are depicted by bright columns. The cut-off of gene-expression changes was 1.50. **A**. Data obtained 3 h after pMCAO for the ischemic rat cortex under Semax treatment; **B**. Data obtained 24 h after pMCAO for the ischemic rat cortex under Semax treatment.

The grouping of the genes according to the molecular functions of their products and to the iReport Web tool revealed that the expression of transcription regulator genes was predominantly enhanced, and that that of genes encoding transmembrane receptors, transport proteins, and various enzymes was decreased 3 h after the onset of ischemia under Semax treatment (Figure 2A); about 39% of the genes with altered expression encoded proteins with molecular functions that were unrelated to the groups presented or were not yet identified. Gene expression was increased mostly at 24 h (Figure 2B). The largest increase in expression was observed for immunoglobulin and cytokine (chiefly chemokine) genes (Table 2). The molecular functions of 24% of the protein products of the genes that exhibited altered expression levels were unknown.

**Table 2:** Genes related to the immune system and exhibited Semax-induced alteration of expression levels

	**Genes related to the immune system (9 genes):**	**Gene symbol**	**ENTREZ GENE_ID**	**Fold change**	**P-value**
**3h**	**MHC (major histocompatibility class) I and II:**				
	RT1 class I, CE15 (RT1-CE15), mRNA.	*RT1-CE15*	414789	0.43	4.0E-06
	RT1 class I, M6, gene 2 (RT1-M6-2), mRNA.	*RT1-M6-2*	365527	0.64	3.0E-03
	RT1 class II, locus Db1 (RT1-Db1), mRNA.	*RT1-Db1*	294270	0.55	7.6E-04
	RT1 class II, locus Ba, mRNA.	*RT1-Ba*	309621	0.51	3.1E-07
	CD74 antigen (invariant polpypeptide of MHC class II antigen-associated), mRNA.	*Cd74*	25599	0.50	1.6E-09
	RT1 class Ia, locus A1, mRNA.	*RT1-A1*	24973	0.50	7.6E-10
	histocompatibility 2, class II antigen E alpha, mRNA.	*H2-Ea*	294269	0.49	4.1E-10
	**Others:**				
	Prostaglandin-endoperoxide synthase 2, mRNA.	*Ptgs2/Cox2*	29527	2.00	8.3E-34
	Intercellular adhesion molecule 1, mRNA.	*Icam1*	25464	1.61	9.9E-05
**24h**	**Genes related to the immune system (36 genes):**				
	**immunoglobulins:**				
	Similar to immunoglobulin heavy chain variable region, mRNA.	*LOC500734*	500734	15.37	7.5E-11
	Similar to immunoglobulin kappa-chain, mRNA.	*LOC500172*	500172	11.57	2.1E-34
	Similar to Immunoglobulin kappa-chain VJ precursor, mRNA.	*LOC500161*	500161	8.31	2.1E-34
	Similar to gamma-2a immunoglobulin heavy chain, mRNA.	*LOC362796*	362796	8.20	2.1E-34
	Similar to immunoglobulin heavy chain variable region, mRNA.	*LOC314492*	314492	6.53	2.0E-06
	Similar to immunoglobulin kappa-chain, mRNA.	*LOC500194*	500194	6.27	2.1E-34
	Similar to Immunoglobulin kappa-chain VJ precursor, mRNA.	*LOC502789*	502789	6.00	3.7E-20
	Similar to immunoglobulin kappa-chain, mRNA.	*LOC502843*	502843	5.74	1.7E-19
	Similar to immunoglobulin kappa-chain, mRNA.	*LOC502797*	502797	5.48	5.5E-11
	Similar to IG kappa-chain V-V region K2 precursor, mRNA.	*LOC500180*	500180	4.67	4.8E-08
	Similar to Igh-1a_predicted protein, mRNA.	*LOC503073*	503073	3.67	7.3E-14
	Similar to NGF-binding Ig light chain, mRNA.	*LOC502820*	502820	3.60	5.6E-23
	Similar to immunoglobulin heavy chain variable region, mRNA.	*LOC500733*	500733	3.08	5.3E-14
	Immunoglobulin heavy chain 1a (serum IgG2a), mRNA.	*Igh-1a*	299352	2.97	3.5E-26
	Similar to NGF-binding Ig light chain, mRNA.	*LOC500183*	500183	2.91	3.8E-22
	Similar to Immunoglobulin kappa-chain VJ precursor, mRNA.	*LOC500162*	500162	2.80	1.7E-04
	Similar to immunoglobulin heavy chain variable region, mRNA.	*LOC503070*	503070	2.70	1.6E-03
	Similar to Immunoglobulin kappa-chain VJ precursor, mRNA.	*LOC500163*	500163	2.62	8.6E-03
	Similar to immunoglobulin light chain variable region, mRNA.	*LOC363828*	363828	2.52	9.7E-03
	Similar to IG light chain Vk region Y13-259, mRNA.	*LOC500181*	500181	1.85	3.2E-07
	Similar to Ig kappa light chain precursor, mRNA.	*LOC500177*	500177	1.76	3.2E-06
	Similar to immunoglobulin light chain variable region, mRNA.	*LOC502831*	502831	0.34	6.3E-06
	**Chemokines:**				
	Chemokine (C-X-C motif) ligand 13, mRNA.	*Cxcl13*	498335	4.12	1.6E-08
	Chemokine (C-X-C motif) ligand 9, mRNA.	*Cxcl9*	246759	2.42	1.8E-14
	Chemokine (C-X-C motif) ligand 10, mRNA.	*Cxcl10*	245920	2.32	1.9E-03
	Chemokine (C-C motif) ligand 5, mRNA.	*Ccl5*	81780	1.98	6.5E-05
	Chemokine (C-X-C motif) ligand 11, mRNA.	*Cxcl11*	305236	1.85	2.1E-03
	Chemokine (C-C motif) ligand 7, mRNA.	*Ccl7*	287561	1.78	8.3E-04
	Chemokine (C-C motif) ligand 19, mRNA.	*Ccl19*	362506	1.72	3.4E-05
	Chemokine (C-C motif) ligand 20, mRNA.	*Ccl20*	29538	0.44	3.4E-05
	**MHC (major histocompatibility class) I and II:**				
	RT1 class II, locus Ba, mRNA.	*RT1-Ba*	309621	2.28	1.5E-15
	RT1 class I, A3, mRNA.	*RT1-A3*	309627	1.96	4.6E-06
	RT1 class Ia, locus A1, mRNA.	*RT1-A1*	24973	1.94	2.8E-11
	RT1 class I, T24, gene 4, mRNA.	*RT1-149*	414784	1.86	3.8E-10
	Histocompatibility 2, M region locus 10.6, mRNA.	*H2-M10.6*	414787	1.77	2.0E-06
	CD74 antigen (invariant polpypeptide of MHC class II antigen-associated), mRNA.	*Cd74*	25599	1.63	5.3E-06

Entrez Gene is NCBI's repository for gene-specific information. In the table, *P*-values are in the form of an exponential number format.

### Biological processes that were significantly associated with the genes that exhibited altered expression levels in response to the administration of the peptide

**Figure 3:**
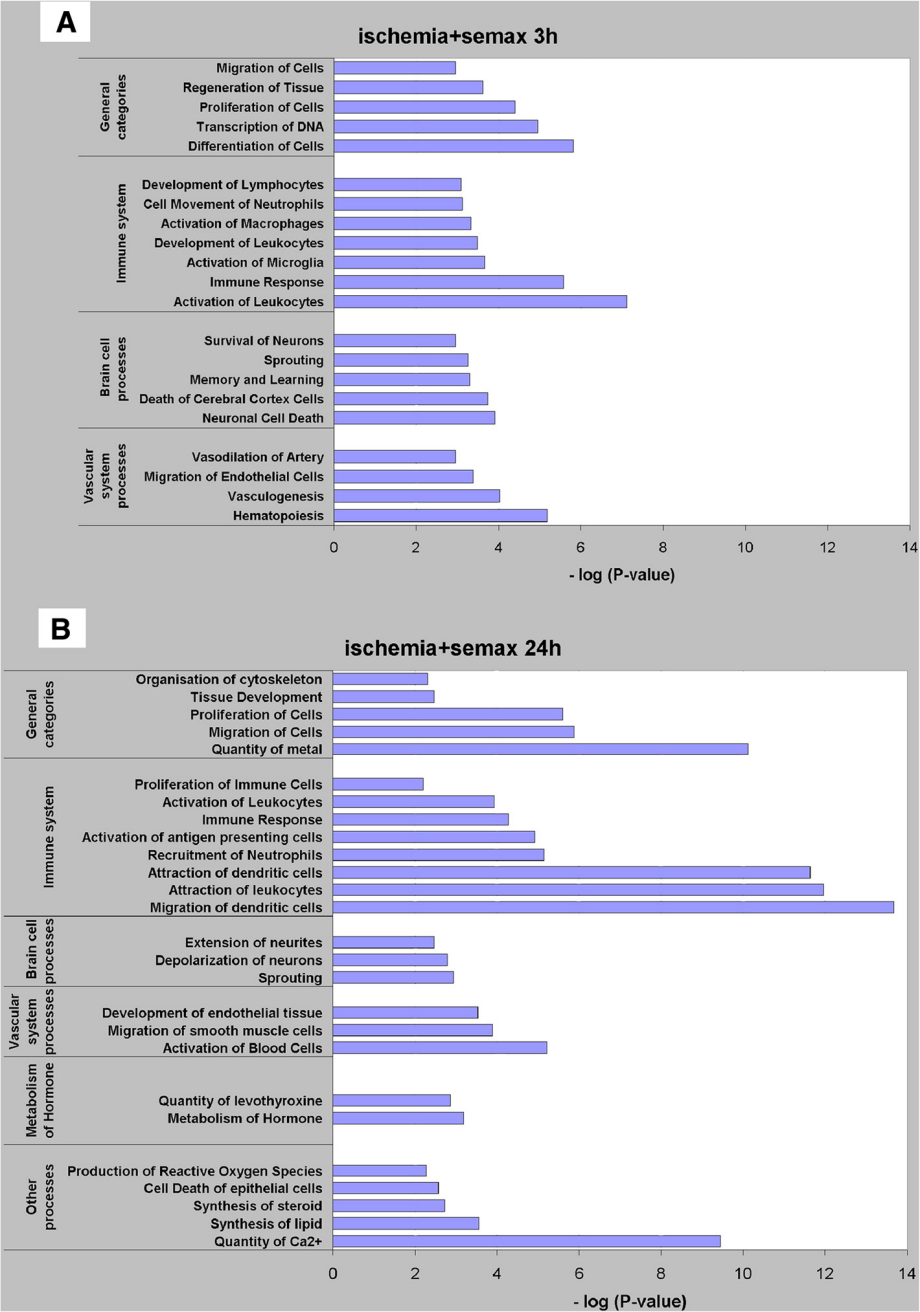
**Biological processes based on the genes that exhibited alteration in their expression levels under Semax treatment.** The x-axis is the absolute value of the log transformed *P*-value, which means that a smaller *P*-value has a larger positive value on the x-axis. Significance was determined using Fisher’s exact test (*P*-value < 0.01). A value of 2 on the x-axis is equivalent to a *P*-value of 0.01. The y-axis shows the categories of biological processes that were related to lists of genes that exhibited changed expression in the experimental conditions. **A**. Data obtained 3 h after pMCAO for the ischemic rat cortex under Semax treatment; **B**. Data obtained 24 h after pMCAO for the ischemic rat cortex under Semax treatment.

We used an online program [12] to analyze genes with altered expression in response to the intermittent administration of Semax to ischemized animals. This led to the identification of several biological processes that were associated with the gene expression changes observed (Figure 3). The reliability of these processes was calculated by Fisher’s exact test.

Three hours after pMCAO, Semax exerted considerable influence on various general biological processes (proliferation, differentiation, and migration of cells), on vascular system processes, brain cell processes, and on the immune system (Figure 3A). Twenty-four hours after occlusion, similar to observed effects 3 h after the procedure, Semax, acting in conditions of focal ischemia, altered the expression of genes involved in cell proliferation and migration. One day after the occlusion, however, unlike at 3 h after the procedure, additional processes supplemented the general processes, namely, the organization of the cytoskeleton, tissue development, and the quantity of metal (Figure 3B). Special attention should be drawn to the processes that were most significantly associated with immune cell activity and calcium ion regulation, namely, the migration and attraction of dendritic cells (DCs), the attraction of leukocytes (Figure 3B), and the regulation of the levels of Ca^2+^ (Figure 3B, Table 3).

**Table 3:** **Genes related to the quantity of Ca**^**2+ **^**and exhibited Semax-induced alteration of expression levels (24 h)**

**Definition (12 genes)**	**Gene symbol**	**ENTREZ GENE_ID**	**Fold change**	**P-value**
Transthyretin (Ttr), mRNA.	*Ttr*	24856	20.55	2.1E-34
Chemokine (C-X-C motif) ligand 13, mRNA.	*Cxcl13*	498335	4.12	1.6E-08
Chemokine (C-X-C motif) ligand 9, mRNA.	*Cxcl9*	246759	2.42	1.8E-14
Chemokine (C-X-C motif) ligand 10, mRNA.	*Cxcl10*	245920	2.32	1.9E-03
Chemokine (C-C motif) ligand 5, mRNA.	*Ccl5*	81780	1.98	6.5E-05
Thyrotropin releasing hormone, mRNA.	*Trh*	25569	1.95	8.5E-08
Chemokine (C-X-C motif) ligand 11, mRNA.	*Cxcl11*	305236	1.85	2.1E-03
Chemokine (C-C motif) ligand 7, mRNA.	*Ccl7*	287561	1.78	8.3E-04
Chemokine (C-C motif) ligand 19, mRNA.	*Ccl19*	362506	1.72	3.4E-05
Secreted phosphoprotein 1, mRNA.	*Spp1*	25353	1.58	3.4E-06
Gastrin releasing peptide, mRNA.	*Grp*	171101	1.53	1.3E-03
Chemokine (C-C motif) ligand 20, mRNA.	*Ccl20*	29538	0.44	3.4E-05

Entrez Gene is NCBI's repository for gene-specific information. In the table, *P*-values are in the form of an exponential number format.

Semax altered the expression of genes related to the immune system to a large degree (Table 2). In conditions of experimental focal ischemia, the action of Semax observed 3 h after the onset of ischemia influenced the expression of several genes that are involved in the regulation of the activity of immune cells: macrophages, neutrophils, and lymphocytes (Figure 3A). The effect of Semax on the immune response was increased significantly 24 h after pMCAO. The genes involved in this process represented over 50% of the total amount of the genes that exhibited altered expression levels. Semax-induced upregulation of transcripts was observed for a majority of the immune-response genes; among these, immunoglobulin genes formed the most prominent group, with half of them exhibiting the highest amplitude of expression alteration among the genes for which the level of transcripts was affected by the peptide (Table 2).

Another remarkable group of genes with Semax-induced alteration in expression levels consisted of genes involved in the vascular system. The expression of 24 and 12 genes was altered 3 and 24 hours after pMCAO, respectively (Table 4).

**Table 4:** Genes related to the vascular system and exhibited Semax-induced alteration of expression levels

	**Genes related to the vascular system (24 genes):**	**Gene symbol**	**ENTREZ GENE_ID**	**Fold change**	**P-value**
**3h**	Cysteine-rich, angiogenic inducer, 61, mRNA.	*Cyr61*	83476	2.43	1.5E-07
	Activating transcription factor 3, mRNA.	*Atf3*	25389	2.24	3.9E-03
	Kruppel-like factor 4 (gut), mRNA.	*Klf4*	114505	2.23	2.0E-03
	A disintegrin-like and metallopeptidse (reprolysin type) with thrombospondin type 1 motif, 1, mRNA.	*Adamts1*	79252	2.18	5.2E-11
	FBJ murine osteosarcoma viral oncogene homolog, mRNA.	*Fos*	314322	2.11	2.0E-04
	Jun B proto-oncogene, mRNA.	*Junb*	24517	2.07	1.8E-06
	Nuclear factor, interleukin 3 regulated, mRNA.	*Nfil3*	114519	2.03	5.4E-08
	Prostaglandin-endoperoxide synthase 2, mRNA.	*Ptgs2/Cox2*	29527	2.00	8.3E-34
	Brain derived neurotrophic factor, mRNA.	*Bdnf*	24225	1.88	2.0E-04
	SWI/SNF related, matrix associated, actin dependent regulator of chromatin, subfamily a, member 5, mRNA.	*Smarca5*	307766	1.74	2.9E-09
	Zinc finger protein 36, mRNA.	*Zfp36*	79426	1.73	5.5E-03
	Early growth response 2, mRNA.	*Egr2*	114090	1.70	6.6E-03
	Dual specificity phosphatase 1, mRNA.	*Dusp1*	114856	1.67	3.0E-08
	Heat shock 27 kDa protein 1, mRNA.	*Hspb1*	24471	1.63	9.0E-03
	Intercellular adhesion molecule 1, mRNA.	*Icam1*	25464	1.61	9.9E-05
	Secretogranin II (chromogranin C), mRNA.	*Scg2*	24765	1.60	3.6E-04
	Relaxin 1, mRNA.	*Rln1*	25616	1.58	4.0E-03
	Amyloid beta (A4) precursor protein, mRNA.	*App*	54226	0.66	1.9E-03
	Mitogen activated protein kinase 10, mRNA.	*Mapk10*	25272	0.66	2.6E-03
	Fibronectin 1, mRNA.	*Fn1*	25661	0.63	5.1E-04
	Catalase, mRNA.	*Cat*	24248	0.51	2.9E-05
	RT1 class II, locus Ba, mRNA.	*RT1-Ba*	309621	0.51	3.1E-07
	CD74 antigen (invariant polpypeptide of MHC class II antigen-associated), mRNA.	*Cd74*	25599	0.50	1.6E-09
	RT1 class Ia, locus A1, mRNA.	*RT1-A1*	24973	0.50	7.6E-10
**24h**	**Genes related to the vascular system (12 genes):**				
	Chemokine (C-X-C motif) ligand 9, mRNA.	*Cxcl9*	246759	2.42	1.8E-14
	Chemokine (C-X-C motif) ligand 10, mRNA.	*Cxcl10*	245920	2.32	1.9E-03
	RT1 class II, locus Ba, mRNA.	*RT1-Ba*	309621	2.28	1.5E-15
	Chemokine (C-C motif) ligand 5, mRNA.	*Ccl5*	81780	1.98	6.5E-05
	RT1 class Ia, locus A1, mRNA.	*RT1-A1*	24973	1.94	2.8E-11
	Chemokine (C-C motif) ligand 7, mRNA.	*Ccl7*	287561	1.78	8.3E-04
	Nephroblastoma overexpressed gene, mRNA.	*Nov*	81526	1.64	3.2E-06
	CD74 antigen (invariant polpypeptide of MHC class II antigen-associated), mRNA.	*Cd74*	25599	1.63	5.3E-06
	Secreted phosphoprotein 1, mRNA.	*Spp1*	25353	1.58	3.4E-06
	Plasminogen activator, tissue, mRNA.	*Plat*	25692	0.65	1.1E-07
	Podoplanin, mRNA.	*Pdpn*	54320	0.63	5.3E-10
	A disintegrin-like and metallopeptidse (reprolysin type) with thrombospondin type 1 motif, 1, mRNA.	*Adamts1*	79252	0.56	7.5E-09

Entrez Gene is NCBI's repository for gene-specific information. In the table, *P*-values are in the form of an exponential number format.

Genes that regulate the levels of Ca^2+^ formed a separate group of genes exhibiting a significant Semax-induced alteration of expression 24 h after occlusion (Table 3).

## Discussion

### Different profiles of gene expression elicited by Semax administration 3 and 24 h after pMCAO

The dynamic state of mRNA expression in mammalian tissues changes during pathophysiological processes and after the introduction of medicinal peptides into the organism. In this context, we studied transcriptome changes caused by the action of neuropeptide Semax in the ischemized rat brain cortex. This genome-wide study showed that Semax affected the transcript level of several dozens of genes 3 and 24 h after pMCAO; however, the functional significance of many of them remains unknown.

Three hours after pMCAO was used in the analysis as a time point inside the therapeutic window of the drug and within the response time of early-response genes [13]. At that time point, we found a considerable alteration of the expression of genes encoding transcription factors that could set off new signal pathways that allow the correction of the destructive processes that developed after vascular occlusion. During the active stage of ischemia and the response of late-response genes, i.e., 24 h after pMCAO, we observed increased levels of transcripts encoding transmembrane receptors and enzymes, especially cytokines and immunoglobulins. One can presume that processes initiated by transcription factors during the first hours of therapy of ischemized animals were developing further. Several similar processes were observed in the course of the associative analysis of biological processes.

### Response of immune system cells to Semax administration and regulation of the expression of genes encoding chemokines and immunoglobulins

The detailed analysis of genes that exhibited altered levels of expression 3 and 24 h after pMCAO allowed the determination of the effect of Semax on various biological processes that were categorized under broad subgroups, namely, general category, brain cell, immune, and vascular processes. The neuroprotective and nootropic properties of Semax were previously associated only with events that are directly relevant to nervous tissues [2,7,11]. Here, we uncovered the action of Semax on the immune system for the first time. Three hours after pMCAO, Semax acted on microglia and immune system cells. The process of leukocyte activation was affected most significantly (P-value = 7.6 × 10^−8^) in the immune response subgroup. The processes that developed 24 h after pMCAO, which involved leukocytes, remained significant. In addition, Semax affected DCs, the presence of which in rat cerebral hemisphere ischemia-damaged tissues had been reported by other researchers [14]. DCs constitute a heterogeneous class of antigen-presenting cells that are capable of immune response initiation [15] and cytokine production [16].

Both inflammation and immune response play an important role in ischemic stroke. It is well known that the penetration of inflammatory/immune cells into brain tissues during the postischemia hours aggravates the situation [17-19]. In addition, no data have been reported to date indicating the presence of a specific cause-and-effect relationship between the penetration of leukocytes into the damaged tissues and the pathogenesis of the ischemia itself [20]. However, some studies support the neuroprotective abilities of immune cells [21,22].

It should be mentioned that the most noticeable immune response to Semax action was observed at 24 h after pMCAO. A high level of immunoglobulin transcripts was found at that time point in the ischemized rat brain cortex. Several studies had shown previously that intravenous immunoglobulin (IVIG) has a strong neuroprotective effect against ischemic impairment of the brain [23]. It is believed that IVIG application is one of the options for acute brain stroke therapy [24]. Whether or not the neuroprotective effect of Semax can be a consequence of the enhancement of the expression of immunoglobulin should be addressed in future studies.

Cytokines (particularly chemokines), which are one of the most important participants in the immune response, were also expressed actively 24 h after pMCAO under the influence of Semax in the region of the brain where the ischemic lesion was localized. Many reports have described chemokine expression in astrocytes, microglia, and even neurons [25]. It is accepted that some chemokines and their receptors are involved in various neurodegenerative diseases [26], including ischemic brain damage [27].

Recent research has shown that chemokines are a unique class of neuromediators that ensure the cross-talk between neurons and cells from their surrounding microenvironment [28]. In accordance with this, the division of chemokines into pro- and anti-inflammatory factors seems to be too simplified and gives rise to contradicting opinions regarding the neuroprotective and neurodegenerative functions of chemokines [29]. Enhanced expression of chemokine-encoding genes is one more evidence in favor of the possible existence of a Semax immunomodulatory effect in conditions of focal cerebral ischemia of the brain.

Semax-induced activation of chemokine genes presumably accounted for the altered transcript level of genes associated with the regulation of the quantity of Ca^2+^ (Figure 3B, Table 3). The ability of some chemokines to raise the level of intracellular Ca^2+^, which plays a messenger role in nervous tissues, has been described in several studies [30,31]. A study that used human neutrophils [32] offered experiment-based support of the effect of Semax on the Ca^2+^ level in cells, and showed an increase in Ca^2+^ levels caused by the effect of Semax on the mechanisms that regulate Ca^2+^-dependent channels.

It is well known that ischemia-induced energy depletion in cells results in disturbed operation of potential-dependent calcium channels and Na^+^/Ca^2+^ pumps, excessive intracellular accumulation of Ca^2+^ ions, and neuronal death [33]. However, it has been shown that Semax contributes to neuron survivability in the conditions of glutamate neurotoxicity that accompany ischemia [3]. Some authors have suggested that cellular death is caused by the Ca^2+^ influx pathway, and not by Ca^2+^ load [34]. Possibly, the neuroprotective effect of Semax on ischemia-damaged nervous tissues includes the impact of Ca^2+^ penetration into the cell on the regulatory processes. This idea is based on recent studies of the neuroprotective effect of Ca^2+^-activated potassium channels in conditions of brain ischemic damage [35,36].

The opinions on the role of the immune system in the pathogenesis of ischemia vary. Studies are available regarding the contribution of the immune system to ischemic damages [37], the neuroprotective and healing effect of immune-cell activation [38,39], the protective role of the immune system, and its therapeutic function [40-42]. It cannot be ruled out that the observed effect of Semax on brain stroke can be explained by its impact on protective immune mechanisms. Some recent reports have described interactions between nervous tissues and the immune system, which were observed after the administration of neuropeptides. For instance, the nootropic medication cerebrolysin favored the survival of immunocompetent cells [43]. Another preparation, the vasoactive intestinal peptide (VIP), which has neurotrophic effects, acted as an immunomodulator [44]. The possible effect of neuromodulation on the consequences of ischemia is believed to be real, although it has not been studied sufficiently [45].

### Response of the vascular system to the administration of the neuropeptide Semax

Here, we found changes in the expression levels of several genes involved in the functioning of the vascular system as a response to Semax administration. The formation of new blood vessels in the ischemized areas represents one of the approaches used in the treatment of brain stroke [46]. It should be mentioned that the presence of immune cells in the damaged tissues is a typical feature of postischemic revascularization [47]. Three hours after pMCAO, Semax affected the expression of genes involved in vasculogenesis and the transcription levels of genes associated with hematopoiesis and the migration of endothelial cells. Some signal pathways are well known to be active in both hematopoiesis and vasculogenesis [48]. Moreover, a large number of genes are expressed in both endothelial cells and hematopoietic precursor cells of the adult organism [49,50]. Three hours after occlusion, Semax altered the expression of genes associated with the artery vasodilation process as well. Our earlier studies showed that capillary bore extension was observed as early as 15 min after the administration of the peptide [9].

As shown in Figure 3B, 24 h after occlusion, Semax affected the development of the endothelial tissue and the migration of smooth muscle cells, which was an indication of vessel formation and stabilization [48]. Finally, another biological process, i.e., the activation of blood cells, was affected by Semax 24 h after pMCAO, which followed logically after the process of the formation of blood cells induced by Semax 3 h after the occlusion.

Thus, as demonstrated here, the action of Semax on the expression of genes that ensure the formation and functioning of the vascular system in ischemic conditions also uncovered its possible vascular and regenerative properties, in addition to its neuroprotective and vasoactive effects.

## Conclusions

In this study, we analyzed the action of the neuroprotective peptide Semax on the transcriptome of rat brain cortical cells in conditions of experimental focal ischemia. Although Semax has been shown to be effective in brain stroke therapy, the molecular mechanisms underlying its neuroprotective action remain unknown.

As shown here, Semax influenced various biological processes that contribute to the functioning of the different systems of the organism. The immune response was most markedly affected by the action of Semax. The peptide increased the amount and mobility of immune cells and enhanced the expression of chemokine and immunoglobulin genes.

Our data showed that Semax is likely to influence processes that accompany the formation of new blood vessels during early ischemia cascade stages, as well as their stabilization at later stages.

The expression of genes responsible for the intracellular level of Ca^2+^ was sensitive to Semax administration against the background of the unfolding pMCAO-induced neurodegenerative processes. Our results showed that Semax enhanced the expression of genes encoding protein products that promote intracellular Ca^2+^ accumulation. Possibly, the neuroprotective effect of Semax on ischemia-damaged nervous tissues includes an impact on processes involved in the incorporation of Ca^2+^ into cells.

Thus, the immunomodulating effects of Semax described here, as well as its influence on the vascular system in conditions of ischemia, are likely to be key factors in the neuroprotective effects of the peptide. It cannot be ruled out that the large amount of genes that exhibited changed levels of expression, the functions of which remain unknown or not well studied, will help disclose other, hitherto unknown pathways of Semax action on damaged brain tissues. We must state at the same time that the baffling complexity of the multicomponent nature of cerebral ischemia and the ability of Semax to affect a large number of biological processes require future research to uncover the full scope of the mechanisms of action of this peptide.

## Methods

### Animals

All experimental protocol were approved by Bioethics Comission of Lomonosov Moscow State University in accordance with the National Institutes of Health Guide for the Care and Use of Laboratory Animals (NIH Publ. no. 80–23, revised 1996). We used adult male Wistar rats (270–320 g) maintained on a 12 h light/dark cycle at a temperature of 22–24°C with free access to food and water.

### Focal cerebral ischemia model

We applied the model of “focal cerebral ischemia” induced as previously described [51]. The irreversible electrical coagulation of the distal segment of the left middle cerebral artery was performed under anesthesia with chloral hydrate (300 mg/kg).

Focal cerebral ischemia was induced by direct pMCAO, involving craniotomy technique as previously described [52] without occlusion of carotid artery. In detail, anesthesia was induced by intraperitoneal administration of chloral hydrate (400 mg/kg body weight). The left middle cerebral artery (MCA) was exposed via the transtemporal approach. A 1.5 cm scalp incision was made at the midpoint between the right eye and the right ear. The temporalis muscle was separated in the plane of its fiber bundles and retracted in order to expose the zygoma and squamosal bone. Using microsurgical technques, a burr hole, 2 mm in diameter, was made with a dental drill 1 mm rostal to the anterior junction of the zygoma and the squamosal bone. The dura mater was carefully pierced with a scalpel. The exposed MCA was isolated and occluded by short coagulation using a bipolar coagulator. The craniotomy was covered with a small piece of gelfoam, the temporalis muscle and overlying skin were allowed to fall back and were sutured separately. After suturing, rats were returned to their cages until sacrifice. The operation was last about 30 min.

### Experimental groups

Animals were divided into two groups: (1) “ischemia” and (2) “ischemia + Semax” groups. pMCAO was performed in all animals. During the experiment, ischemia + Semax animals were given intraperitoneal injections of Semax (100 μg/kg), whereas ischemia animals were injected with saline. The injections of Semax or saline were performed 15 min, 1, 4 and 8 h after pMCAO.

The rats were decapitated under anesthesia with ethyl ether 3 and 24 h after the operation. According to data from the literature, significant events in the formation of a stroke area, such as excitotoxicity, mitochondrial damage, emergence of reactive oxygen species, and apoptosis, occur within the first 3 h after occlusion of an artery [53], and the expression of genes at the early stage of ischemia can be studied at this time point. At the 24 h time point the infarction area reaches its maximal dimensions and the formation of the penumbra is completed [54].

Each time point included at least five animals. We isolated the frontoparietal cortex of the ischemic animals, in which, according to histological analysis of our earlier research, the damaged area was localized [51]. Total RNA was isolated from tissue samples.

### Microarray data analysis

Microarray experiments were carried out at ZAO ''Genoanalytica'', Moscow, Russia. Total RNA was isolated from tissue samples using guanidine thiocyanate [55]. RNA integrity was assessed by comparison with the rRNA bands obtained in agarose gel electrophoresis under denaturing conditions. RNA was quantified using NanoDrop, and its quality was assessed using an Agilent RNA 6000 Nano Chip. Total RNA (400 ng) was amplified using an Illumina® TotalPrep™ RNA Amplification Kit (Ambion, USA) containing 22,523 probes for a total of 22,228 rat genes selected primarily from the NCBI Reference Sequence database (Illumina, USA). The Illumina RatRef-12 Expression BeadChip was used in accordance with the manufacturer’s instructions. The BeadArray Reader was employed for data acquisition, and the analysis was accomplished with the help of the Genome Studio software (Illumina, USA) using the gene-expression module. The statistical algorithm used in GenomeStudio gene expression analysis is the Illumina Custom error model.

### Functional analysis

The interactive Web-based Ingenuity iReport program [12] based on Fisher’s exact test (*P*-value < 0.01) was applied to identify the molecular functions of the products of the genes that exhibited altered expression in the conditions established, as well as signaling pathways and statistically significant biological processes. Ingenuity iReport helps the quick identification of especially significant genes, signaling pathways, and processes that are most relevant to the experimental data. Only those genes with a change in expression of at least 1.5-fold from the baseline value and whose *P*-value lower 0.05 were selected for iReport analysis.

### Availability of supporting data

The data sets supporting the results of this article are available in the ArrayExpress repository (European Bioinformatics Institute, Cambridge, UK) [56] with series accession number E-MTAB-1864 (https://www.ebi.ac.uk/biosamples/group/SAMEG148775).

## Abbreviations

ACTH: Adrenocorticotropic hormone; pMCAO: Permanent middle cerebral artery occlusion; MCA: Middle cerebral artery; CNS: Central nervous system; DC: Dendritic cells; IVIG: Intravenous immunoglobulin; VIP: Vasoactive intestinal peptide; MHC I and II: Major histocompatibility class I and II.

## Competing interests

The authors declare that they have no competing interests.

## Authors’ contributions

EM - carried out the molecular genetic studies and drafted the manuscript; NM - synthesized Semaks and participated in the design of the study; VS - designed the focal ischemia model; OP - performed the operations of experimental animals; VD - isolated the frontoparietal cortex of the ischemic animals, obtained the RNA from the tissue samples; LD – have made substantial contributions to interpretation of data and have been involved in revising manuscript critically for content; SL - have given final approval of the version to be published. All authors read and approved the final manuscript.

## Supplementary Material

Additional file 1: Table S1 List of all genes that exhibited changed expression under Semax treatment (3 h after pMCAO). All transcripts that showed significant difference between the “ischemia + Semax” and “ischemia” animal groups 3 h after pMCAO. In the table, *P*-values are in the form of an exponential number format. Entrez Gene is NCBI’s repository for gene-specific information. Click here for file

Additional file 2: Table S2 List of all genes that exhibited changed expression under Semax treatment (24 h after pMCAO). All transcripts that showed significant difference between the “ischemia + Semax” and “ischemia” animal groups 3 h after pMCAO. In the table, *P*-values are in the form of an exponential number format. Entrez Gene is NCBI’s repository for gene-specific information. Click here for file
